# Impact of Sleeve Gastrectomy and Roux-en-Y Gastric Bypass on Esophageal Physiology and Gastroesophageal Reflux Disease: A Prospective Study

**DOI:** 10.1007/s11695-025-07818-4

**Published:** 2025-04-14

**Authors:** Ahmed Mohammed Farid Mahmoud Mansour, Abd El Hamid Ahmed Ghazal, Mohamed Ibrahim Kassem, Elettra Ugliono, Mario Morino, Mostafa Refaie ElKeleny

**Affiliations:** 1 Alexandria University, Alexandria, Egypt; 2 University of Turin, Turin, Italy

**Keywords:** Sleeve gastrectomy, Roux-en-Y gastric bypass, Esophagus, Pathophysiology, Gastroesophageal reflux disease, 24-h pH-impedance monitoring, Esophageal manometry

## Abstract

**Background:**

Laparoscopic sleeve gastrectomy (LSG) and laparoscopic Roux-en-Y gastric bypass (LRYGB) are the most commonly performed bariatric surgical procedures. The effectiveness of these operations on weight control is well established; however, their impact on esophageal physiology is still under evaluation. The aim of this study is to evaluate the consequences of LSG and LRYGB on esophageal physiology, especially concerning reflux.

**Methods:**

This prospective study involved 30 patients with severe obesity; 15 underwent LSG, and 15 had LRYGB. Conducted between 2021 and 2023 in Turin, Italy, the study employed preoperative and 1-year postoperative assessments of esophageal function using conventional esophageal manometry, 24-h multichannel intraluminal impedance-pH (MII-pH), upper gastrointestinal series, upper endoscopy, and a validated questionnaire to assess outcomes related to esophageal and lower esophageal sphincter (LES) functions and reflux.

**Results:**

Both groups experienced significant reductions in weight and body mass index, with *p*-values < 0.001 for both measures. The LRYGB group achieved a significantly higher percentage of excess weight loss compared to the LSG group, with a *p*-value of < 0.001. In the LSG group, GERD symptoms remained unchanged postoperatively (*p* = 0.687), with 26.7% using proton pump inhibitors (PPIs) before and after surgery, while in the LRYGB group, GERD symptoms and PPIs use significantly decreased from 53.3 to 6.7% (*p* = 0.016). Quality of life improved significantly in both groups, with a *p*-value of 0.001. In the LRYGB group only, esophagitis significantly decreased from 53.3 to 6.7% (*p* = 0.007), and barium studies showed a significant reduction in reflux signs from 66.7% preoperatively to none postoperatively (*p* = 0.002). Multichannel intraluminal impedance-pH monitoring revealed significant reductions in reflux metrics for LRYGB group only: total refluxes decreased from 29.0 to 15.0, acidic refluxes from 12.0 to 8.0, and the DeMeester score from 4.70 to 3.70 (*p* = 0.026, 0.033, and 0.029, respectively). Regarding the manometric parameters, significant changes were observed in the LSG group: total LES length decreased from 34.0 to 31.33 mm (*p* = 0.027) and residual pressure increased from 2.0 to 4.0 mmHg (*p* = 0.012), also peristaltic wave amplitude decreased from 98.20 to 52.93 mmHg (*p* < 0.001), while in the LRYGB group, only the LES residual pressure significantly increased from 2.0 to 4.0 mmHg (*p* = 0.006).

**Conclusions:**

LSG and LRYGB are effective for weight loss and improving quality of life. Sleeve gastrectomy controls reflux, with new cases being rare. Advanced diagnostics are key when standard tests are insufficient.

**Graphical Abstract:**

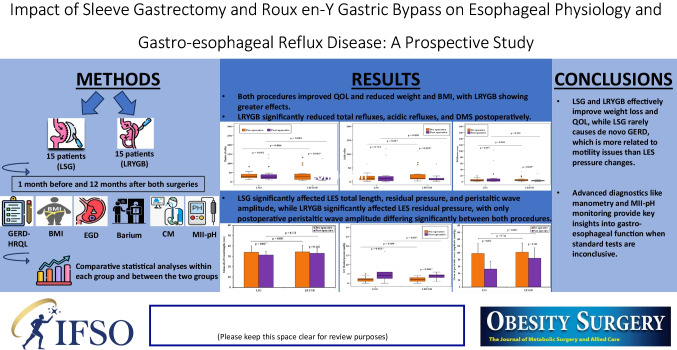

## Introduction

Obesity has escalated into a global health crisis, significantly increasing the incidence of serious conditions such as type 2 diabetes, various cancers, cardiovascular diseases, and gastroesophageal reflux disease (GERD). Bariatric surgery, particularly laparoscopic sleeve gastrectomy (LSG) and laparoscopic Roux-en-Y gastric bypass (LRYGB), remains the most effective treatment for severe obesity, offering significant and long-term weight loss [1]. LSG has gained popularity due to its simplicity, while LRYGB which is more technically demanding, combines restrictive and malabsorptive mechanisms for weight reduction. Although these procedures are widely performed, each carries potential complications that can significantly affect esophageal function and overall quality of life (QOL) [2, 3].

The relationship between LSG and GERD is complex, influenced by various anatomical and physiological factors. Studies have reported mixed outcomes regarding GERD following LSG. Some research indicates an increased incidence of GERD post-LSG, potentially due to factors such as a weakened lower esophageal sphincter (LES) with decreased pressure, disruption of the angle of His, reduced gastric compliance, increased intragastric pressure, delayed gastric emptying, late sleeve dilation, and the development of hiatal hernias [4]. Postoperative improvements in reflux have been observed through mechanisms such as weight loss, which leads to decreased intra-abdominal pressure, reduced gastric acid production, accelerated gastric emptying, and reinforcement of the gastroesophageal sphincter [5]. The varying results highlight the need for a meticulous understanding of LSG’s effects on GERD.

LRYGB is widely regarded as the superior option for managing GERD in patients with severe obesity. Its anatomical changes reduce acid secretion from the small gastric pouch and divert bile, providing significant relief from GERD. The weight loss and the lowering of intra-abdominal pressure achieved with LRYGB further contribute to symptom improvement. Although de novo GERD can occur after LRYGB, it is less frequent than with LSG. When reflux does arise after LRYGB, it is typically due to complications such as fistulas, strictures, or enlarged gastric pouches. These combined factors make LRYGB more effective than LSG in treating GERD [6].

This study aims to compare the effects of LSG and LRYGB on esophageal function and the development of GERD in patients with severe obesity. To achieve this, we utilized conventional esophageal manometry, multichannel intraluminal impedance-pH (MII-pH) monitoring, upper endoscopy, gastrointestinal series, and a validated questionnaire. The findings will contribute to optimizing surgical management strategies for these patients.

## Methods

This prospective, non-randomized, observational study was conducted at a leading bariatric surgery center in Molinette Hospital, Turin, Italy, from January 2021 to June 2023. The study included 30 patients with severe obesity, divided into two groups of 15 each. Group A underwent LSG, while Group B had LRYGB. The study included participants aged 18 to 65 with a body mass index (BMI) over 40 kg/m^2^ or over 35 kg/m^2^ accompanied by related health conditions. Exclusion criteria encompassed individuals with severe GERD, large hiatal hernias, prior major abdominal surgeries, psychiatric disorders, or malignancies. Ethical approval was secured, and all participants provided informed written consent.

Patient management was guided by the Lyon Consensus 2.0 criteria, incorporating both subjective assessment and available objective diagnostic methods to ensure a standardized and evidence-based evaluation of GERD [7].

One month prior to surgery, each patient underwent a comprehensive clinical evaluation. This included gathering a detailed medical history and assessing GERD symptoms using the Gastroesophageal Reflux Disease-Health-Related Quality of Life (GERD-HRQL) questionnaire [8]. A physical examination was conducted to record height, weight, and BMI. Additionally, all patients underwent esophagogastroduodenoscopy (EGD) to assess esophageal and gastric conditions and a barium study to detect the presence of hiatal hernia and evaluate gastric anatomy.

A 24-h MII-pH monitoring was conducted using a portable data logger (Digitrapper pH-Z) and VersaFlex Z disposable pH/impedance catheters (Sierra Scientific Instruments). Patients discontinued all anti-reflux medications for at least 15 days prior to the test. The catheter was inserted transnasally, positioning its distal sensor 5 cm above the upper border of the LES. During the monitoring period, patients recorded their meals, symptoms, and posture changes. The collected data provided a comprehensive analysis of acid, weakly acidic, and non-acid reflux episodes, detailing their frequency, duration, and clearance time. Reflux patterns were evaluated using the DeMeester score (DMS), with a score below 14.72 considered normal.

Esophageal conventional manometry (CM) was performed to assess esophageal motility and LES function. A “Dynograph R-611” motility machine (Beckmann Inc., Germany) system with eight channels perfusion catheters, four disposed radially and oriented at 90° to each other and four positioned longitudinally at intervals of 5 cm being perfused by distilled water (rate of 0.5 ml/min) by the Arndorfer water perfusion system (AMS, Greendale, WI, USA), was used for this purpose. The catheter was positioned through the patient’s nostril, and the patient was semi-seated during the test. Key measurements included LES pressure, total and abdominal LES length, and the duration of LES relaxation. Esophageal peristalsis was evaluated by measuring peak peristaltic pressures and the velocity of esophageal contractions. Data from the manometric studies were analyzed using specialized (SyneticsR, USA) software.

The two surgical procedures were performed by the same experienced surgical team. As described before [9, 10], LSG involved resecting a substantial portion of the stomach along the greater curvature, with particular attention to utilizing a 38Fr bougie and initiating stapling 2 cm from the pylorus, while LRYGB included the creation of a small gastric pouch measuring 20–30 ml, followed by a Roux-en-Y reconstruction with a biliopancreatic limb of 100 cm, a Roux limb of 150 cm, and a gastrojejunostomy measuring 2.5 cm, effectively bypassing a significant portion of the stomach and intestine.

Following surgery, patients underwent evaluations at the 12-month mark to monitor changes in weight, GERD symptoms, and esophageal function. Assessments included the GERD-HRQL questionnaire, EGD, barium swallow study, MII-pH monitoring, and esophageal manometry. The percentage of excess weight loss (PEWL) was calculated, and postoperative weight and BMI data were collected. Statistical analyses were performed using SPSS software, employing parametric or non-parametric tests based on data distribution. Chi-square tests facilitated qualitative comparisons, with significance defined as *p* < 0.05.

## Results

### Clinical Results

From 1 January 2021, to 30 June 2023, this study was conducted involving 15 patients who underwent LSG at The Center for Minimally Invasive Surgery at Molinette Hospital. The LSG cohort comprised 12 females (80%) and 3 males (20%), with a mean age of 46.0 ± 7.38 years. This group was compared with 15 patients who underwent LRYGB, consisting of 14 females (93.3%) and 1 male (6.7%), with a mean age of 47.0 ± 9.89 years. Both groups were evaluated 1 month before surgery and at a median of 12 (11–12) months postoperatively.

In the LSG group, patients had an average preoperative weight of 134.4 kg ± 25.87 kg and a BMI of 49.45 kg/m^2^ ± 6.94 kg/m^2^. After surgery, their weight significantly decreased to an average of 106.7 kg ± 21.33 kg, and their BMI dropped to 39.23 kg/m^2^ ± 5.60 kg/m^2^, with both reductions showing *p*-values < 0.001. In the LRYGB group, preoperative weight was 122.6 ± 18.44 kg and BMI was 44.52 ± 5.50 kg/m^2^. These decreased significantly postoperatively to 91.53 ± 15.56 kg and 33.29 ± 5.25 kg/m^2^, respectively, with *p*-values < 0.001. Although preoperative weight and BMI were similar between the groups (*p*-values 0.161 and 0.054, respectively), postoperative values were significantly lower in the LRYGB group (*p*-values 0.035 and 0.006, respectively). The PEWL was significantly higher in the LRYGB group (50.48 ± 8.13%) compared to the LSG group (36.21 ± 10.64%) with a *p*-value < 0.001.

Prior to surgery, four patients (26.7%) in the LSG group experienced symptoms of GERD. After the procedure, two patients (13.3%) reported complete resolution of their symptoms, two (13.3%) continued to experience symptoms, and two (13.3%) developed new GERD symptoms. The use of full doses of proton pump inhibitors (PPIs) decreased from six patients (40%) preoperatively to four patients (26.7%) postoperatively, with no statistically significant difference (*p*-value 0.687). In the LRYGB group, GERD symptoms were present in eight cases (53.3%) preoperatively and decreased significantly to one case (6.7%) postoperatively (*p*-value 0.016), with PPIs use also significantly decreasing from eight cases (53.3%) to one case (6.7%) (*p*-value 0.016). No significant differences were observed between the groups in GERD symptoms or PPI usage both pre- and postoperatively.

QOL assessed using the GERD-HRQL questionnaire showed significant improvement after both operations. In the LSG group, the median preoperative score was 58.0 (43.0–60.0), and the postoperative score was 25.0 (15.5–30.0), with a *p*-value of 0.001. The LRYGB group had a median preoperative score of 58.0 (44.5–60.5) and a postoperative score of 25.0 (16.5–29.5) with a *p*-value of 0.001. No significant difference was observed between the two groups in QOL scores.

### Diagnostic Results

Preoperative endoscopic evaluations revealed *Helicobacter pylori* (*H. pylori*) infection with the rapid urease test (RUT) in two cases (13.3%) and esophagitis in ten cases (66.7%) in the LSG group, with no instances of Barrett’s metaplasia. Postoperatively, esophagitis was present in four cases (26.7%), and Barrett’s metaplasia was observed in one case (6.7%). In the LRYGB group, one case (6.7%) had *H. pylori* infection with the RUT, and esophagitis was found in eight cases (53.3%) preoperatively, with no Barrett’s metaplasia. Postoperatively, esophagitis was observed in only one case (6.7%) with no Barrett’s metaplasia. All patients who tested positive for RUT in both groups received the appropriate eradication therapy for *H. pylori* prior to surgery, and all tested negative for RUT before the operation and throughout the follow-up period. The differences between groups were not significant.

Barium studies revealed no significant changes in the LSG group before and after surgery. One patient (6.7%) developed esophageal dilation postoperatively. Preoperatively, reflux was observed in five cases (33.3%), which decreased to two cases (13.3%) postoperatively. Small hiatal hernias were present in six cases (40%) before surgery and in three cases (20%) after surgery. All patients exhibited normal stomach/sleeve configurations and duodenal emptying. In the LRYGB group, reflux signs significantly decreased postoperatively as it was evident in ten cases (66.7%) before the operation and disappeared after (*p*-value 0.002). Otherwise, there were no significant differences between the rest studied parameters. Small hiatal hernia was detected in six cases (40%) preoperatively, while postoperatively only two cases (13.3%) had small hiatal hernias. There were no cases of esophageal dilatation nor tortuosity. All cases had normal configuration of the stomach/gastric pouch and jejunal emptying. Both groups had similar findings in barium study parameters.

### Functional Results

In group A, MII-pHmetry data revealed no significant changes before and after surgery in the total number of reflux episodes, nor in the counts of acidic, weakly acidic, non-acidic, and mixed reflux events. Additionally, there were no notable alterations in acid exposure time (AET), bolus exposure time, or DeMeester score (DMS). Prior to surgery, one patient (6.7%) exhibited an increase in the total number of reflux episodes, acidic reflux events, AET%, and DMS beyond normal ranges. Another patient (6.7%) had elevated weakly acidic reflux episodes. Bolus exposure time remained within normal limits for all patients. Post-surgery, three patients (20%) experienced an increase in total reflux events, bolus exposure time%, and DMS beyond normal limits. Among these, one patient had increased acidic reflux episodes, another had increased weakly acidic reflux episodes, and one had both. Overall, two patients (13.3%) had elevated acidic reflux episodes, while three patients (20%) had increased weakly acidic reflux episodes, resulting in a total of seven patients (46.7%) with an increased AET%.

**Fig. 1:**
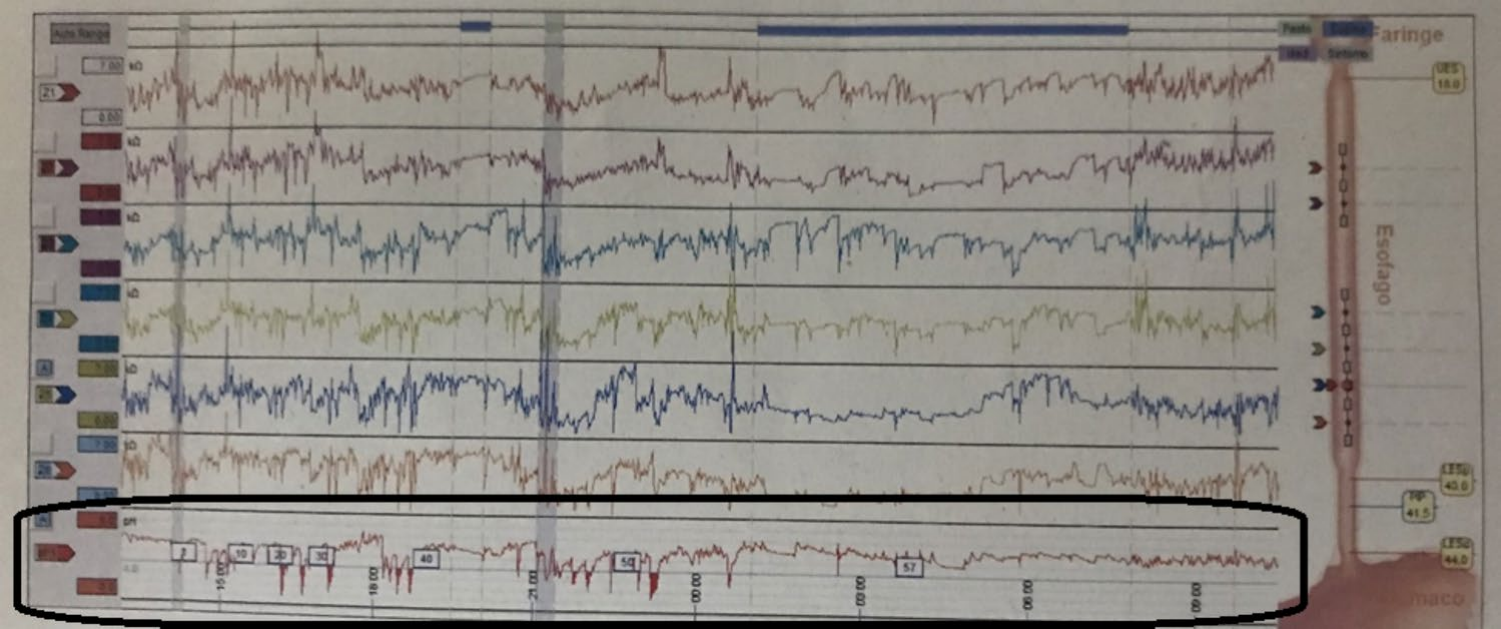
MII-pH with few short attacks of acidic refluxes specially during the daytime in a case after LRYGB

In group B, there were statistically significant decreases postoperatively in the total number of refluxes, acidic refluxes, and DMS. The median total number of refluxes dropped from 29.0 (12.0–42.5) to 15.0 (12.50–22.50), acidic refluxes decreased from 12.0 (10.0–26.5) to 8.0 (6.50–13.0), and DMS from 4.70 (3.30–8.15) to 3.70 (2.45–4.45), with *p*-values of 0.026, 0.033, and 0.029, respectively. The MII-pH of a case with few short attacks of acidic refluxes (specially during the daytime) after LRYGB is shown in Fig. 1.

Despite these improvements, there were no significant differences in weakly acidic, non-acidic, mixed refluxes, AET%, or bolus exposure time postoperatively. Preoperatively, one case (6.7%) had increased weakly acidic refluxes and bolus exposure time%, while three cases (20%) had elevated AET%. After surgery, only one case (6.7%) continued to show increased weakly acidic refluxes, bolus exposure time%, and AET%.

There were no significant differences between group A and group B, both before and after surgery, in any of the measured reflux parameters which are summarized in Table 1.

**Table 1:** Comparison between the two studied groups according to MII-pH

MII-pH	LSG (*n* = 15)		LRYGB (*n* = 15)		*p*1	*p*2
	Preoperative	Postoperative	Preoperative	Postoperative		
Number of refluxes						
Min.–Max	12.0–82.0	10.0–146.0	6.0–132.0	8.0–126.0	0.806	0.081
Median (IQR)	28.0 (21.5–41.5)	25.0 (16.50–39.0)	29.0 (12.0–42.5)	15.0 (12.50–22.50)		
*p*	0.691		0.026*			
Acid exposure time%						
Min.–Max	0.20–6.80	0.20–29.30	0.90–6.80	0.80–9.10	0.775	0.325
Median (IQR)	2.10 (1.65–2.70)	2.40 (1.70–7.10)	2.10 (1.65–2.95)	2.30 (1.55–2.75)		
*p*	0.124		1.000			
Acidic reflux						
Min.–Max	2.0–65.0	0.0–120.0	3.0–111.0	2.0–64.0	0.267	0.838
Median (IQR)	12.0 (6.50–20.5)	11.0 (3.0–19.50)	12.0 (10.0–26.5)	8.0 (6.50–13.0)		
*p*	0.712		0.033*			
Weak acidic reflux						
Min.–Max	2.0–41.0	2.0–65.0	0.0–37.0	0.0–62.0	0.250	0.305
Median (IQR)	16.0 (6.50–21.0)	8.0 (5.0–26.50)	5.0 (2.50–23.0)	8.0 (4.50–10.0)		
*p*	0.865		0.929			
Non-acidic reflux						
Min.–Max	0.0–6.0	0.0–4.0	0.0–6.0	0.0–0.0	1.000	0.539
Median (IQR)	0.0 (0.0–0.0)	0.0 (0.0–0.0)	0.0 (0.0–0.0)	0.0 (0.0–0.0)		
*p*	0.416		0.109			
Mixed reflux						
Min.–Max	0.0–28.0	0.0–46.0	0.0–65.0	2.0–31.0	0.624	0.683
Median (IQR)	9.0 (7.50–14.50)	8.0 (3.0–18.50)	10.0 (3.0–11.50)	10.0 (6.0–12.0)		
*p*	0.733		0.925			
Bolus exposure time%						
Min.–Max	0.20–2.10	0.20–16.10	0.20–2.60	1.0–4.20	0.461	0.436
Median (IQR)	1.60 (1.20–1.70)	1.70 (1.20–2.35)	1.70 (1.15–2.0)	1.80 (1.55–1.95)		
*p*	0.195		0.093			
DeMeester score						
Min.–Max	0.60–18.90	0.60–103.1	0.70–33.40	0.40–26.50	0.624	0.539
Median (IQR)	4.70 (2.15–7.60)	4.90 (1.75–9.80)	4.70 (3.30–8.15)	3.70 (2.45–4.45)		
*p*	0.397		0.029*			

*IQR* interquartile range, *Min.* minimum, *Max.* maximum, *p1 p*-value for comparing between the two studied groups for preoperative parameters, *p2 p*-value for comparing between the two studied groups for postoperative parameters

*Statistically significant at *p* ≤ 0.05

**Fig. 2:**
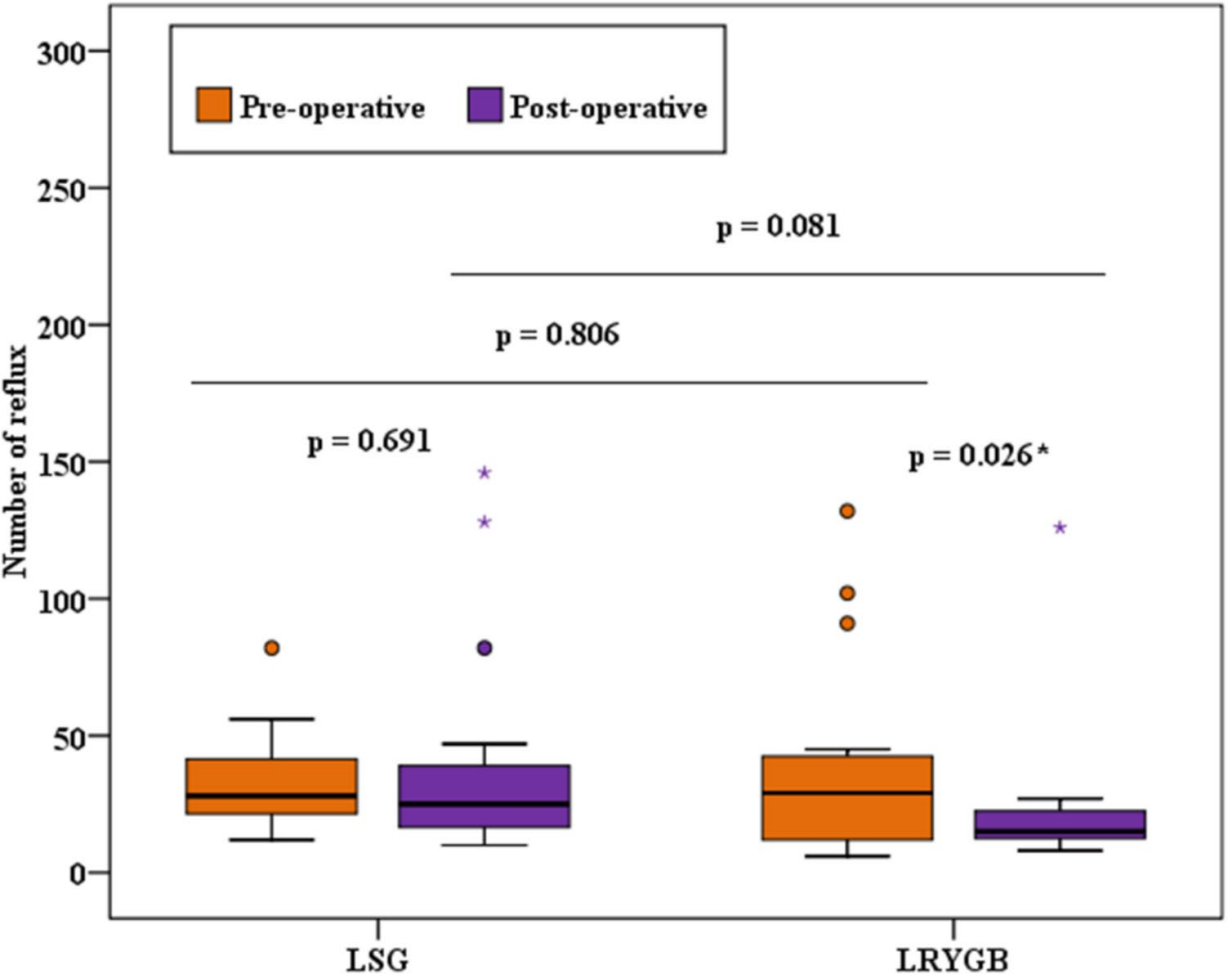
Comparison between the two studied groups according to the number of refluxes

**Fig. 3:**
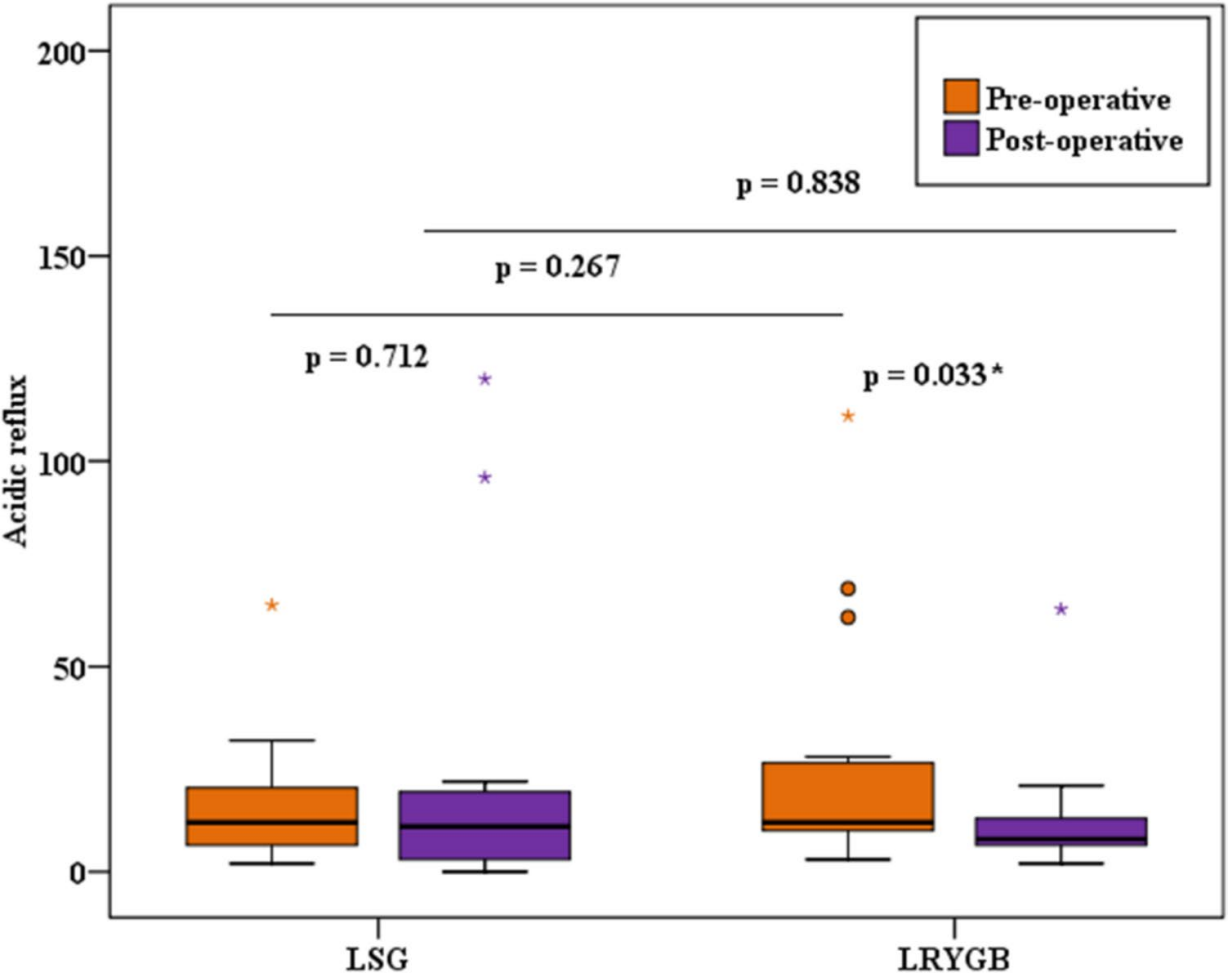
Comparison between the two studied groups according to the number of acidic refluxes

**Fig. 4:**
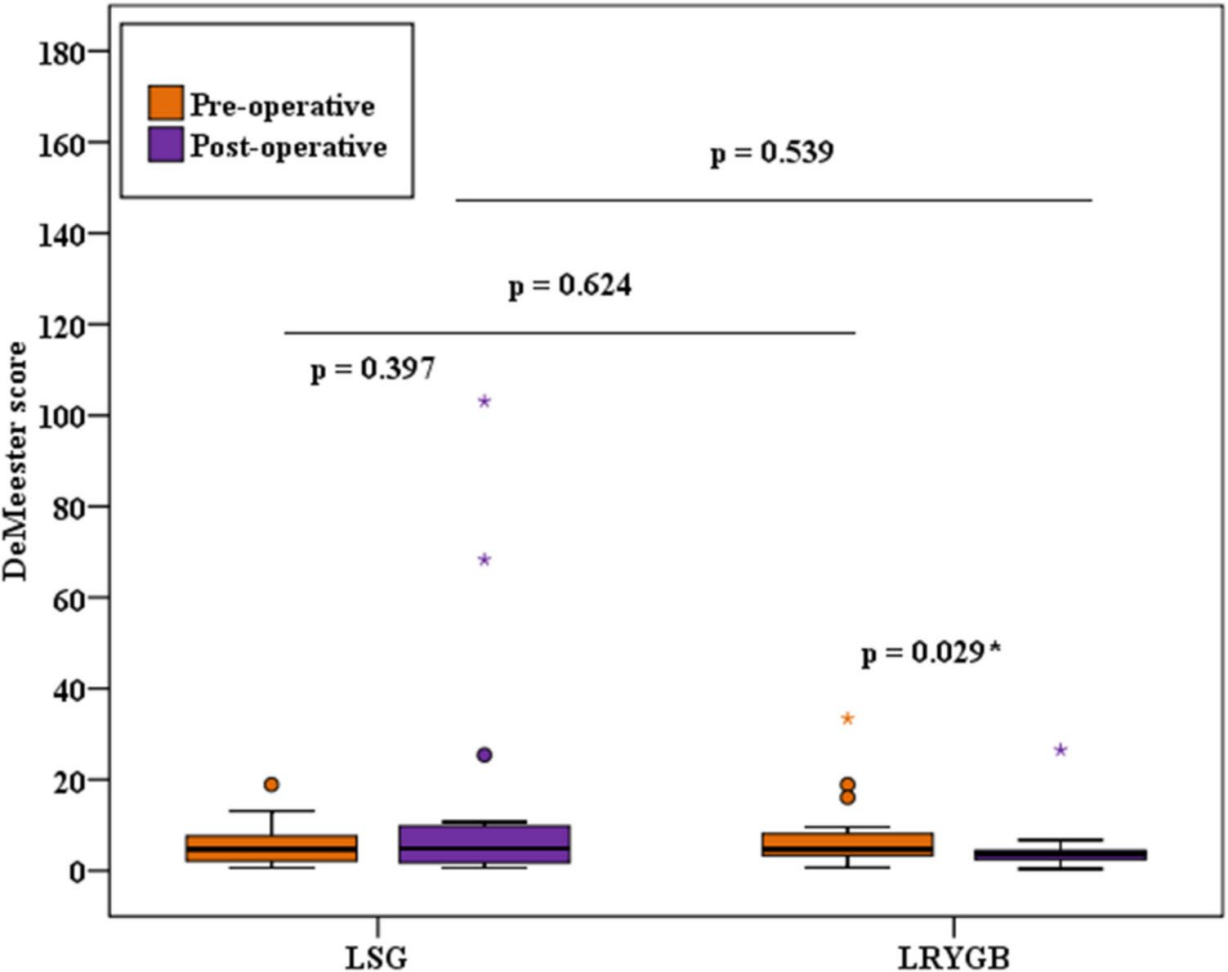
Comparison between the two studied groups according to DeMeester score

The comparisons between both groups regarding the total number of refluxes, acidic refluxes, and DMS are shown in Figs. 2, 3, and 4.

**Fig. 5:**
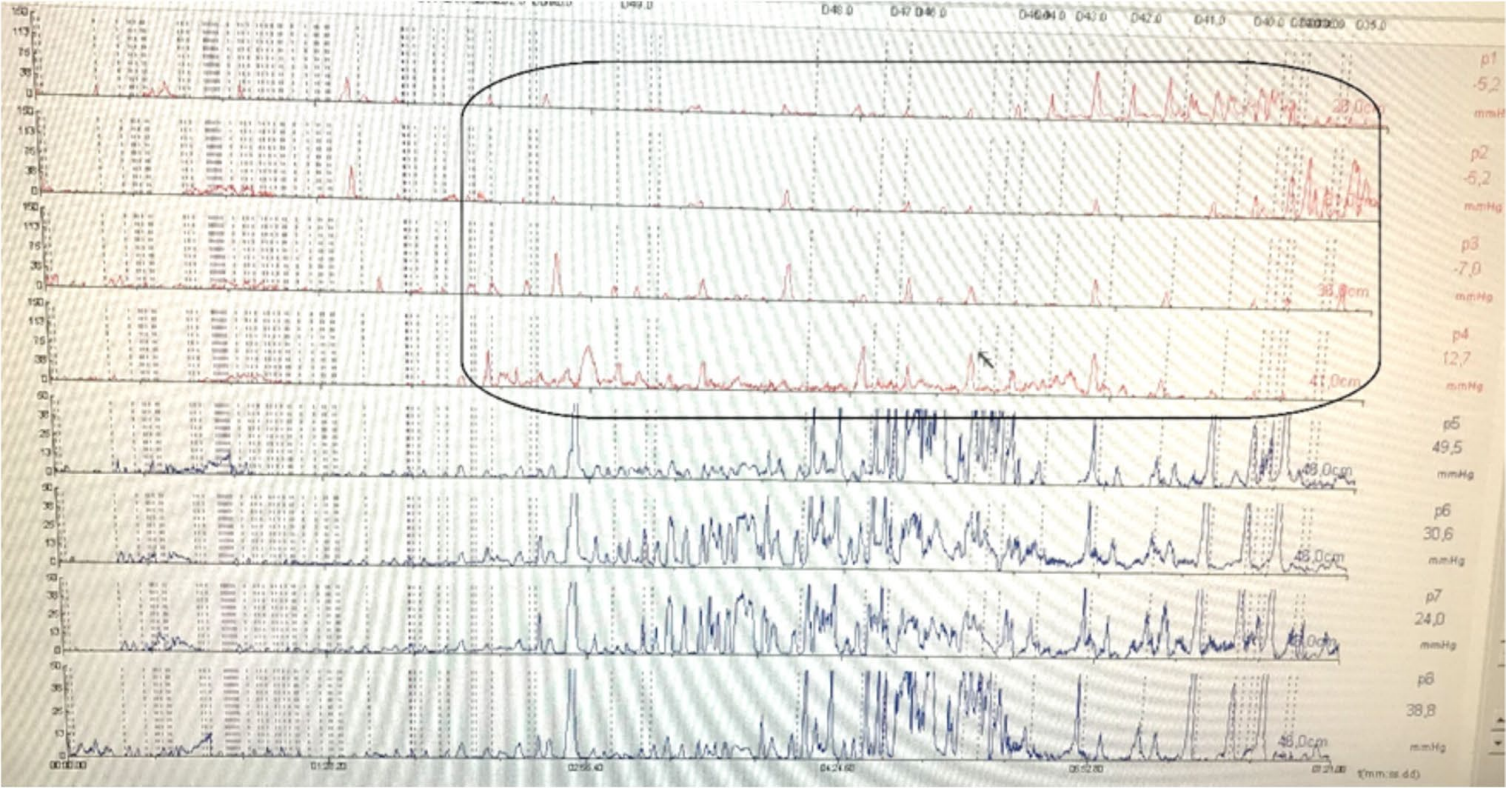
Weak peristaltic waves’ amplitude in a case after LSG

Also in group A, CM revealed significant changes in LES total length, LES residual pressure, and peristaltic wave amplitude before and after surgery. Figure 5 shows a CM of a group A case with weak peristaltic waves’ amplitude postoperatively.

The mean length of the LES decreased from 34.0 mm (± 6.04) to 31.33 mm (± 3.52), with one case (6.7%) falling below the normal range both before and after surgery. The median LES residual pressure increased from 2.0 mmHg (1.0–2.50) to 4.0 mmHg (2.50–6.0), with only one case (6.7%) exceeding the normal range postoperatively. Additionally, the mean peristaltic wave amplitude significantly decreased from 98.20 mmHg (± 29.34) to 52.93 mmHg (± 22.60), with one case (6.7%) falling below the normal range after surgery. The *p*-values for these changes were 0.027, 0.012, and < 0.001, respectively.

Other LES metrics, such as basal pressure, abdominal length, percentage of complete relaxations, and duration of relaxations, showed no significant changes postoperatively. Similarly, no significant changes were observed in peristaltic wave characteristics like percentage of normal morphology and velocity. Preoperatively, five cases (33.3%) had LES basal pressure below the normal range, increasing to six cases (40%) postoperatively. LES abdominal length was below normal in six cases (40%) pre-surgery, improving to three cases (20%) postoperatively. One case (6.7%) showed failure of complete relaxations postoperatively, while all cases had normal relaxation duration before and after surgery. Peristaltic waves were generally normal in morphology and velocity, except for one case (6.7%) after surgery. One patient (6.7%) developed type II achalasia post-surgery, with a basal LES pressure of 41 mmHg, residual pressure of 17 mmHg, failure of complete relaxations, and absent peristaltic waves, preventing further assessment.

While in group B, the only significant change observed was in the median LES residual pressure, which increased from 2.0 (1.0–3.0) mmHg to 4.0 (3.0–4.50) mmHg postoperatively, with all cases remaining within the normal range. The *p*-value for this change was 0.006.

No significant changes were noted in LES basal pressure, total length, abdominal length, complete relaxations%, duration of relaxations, or peristaltic wave characteristics, including amplitude, normal morphology%, and velocity. Preoperatively, two cases (13.3%) had LES basal pressure below the normal range, which reduced to one case (6.7%) postoperatively. LES total length was below normal in one case (6.7%) pre-surgery, and LES abdominal length was below normal in six cases (40%) pre-surgery, improving to one case (6.7%) postoperatively. All cases in group B exhibited normal LES complete relaxations, duration of relaxation, and residual pressure before and after surgery. Peristaltic wave characteristics remained normal both pre- and postoperatively.

Comparing the two groups, the only significant difference between them was in the postoperative peristaltic wave amplitude (*p*-value 0.003), with no other significant differences in any of the CM parameters measured, as summarized in Table 2.

**Table 2:** Comparison between the two studied groups according to conventional manometry

CM	LSG (*n* = 15)		LRYGB (*n* = 15)		*p*1	*p*2
	Preoperative	Postoperative	Preoperative	Postoperative		
LES basal pressure (mmHg)						
Min.–Max	6.0–25.0	6.0–41.0	6.0–24.0	11.0–22.0	0.539	0.187
Median (IQR)	14.0 (9.0–17.50)	14.0 (8.0–17.0)	15.0 (13.0–18.5)	15.0 (13.0–19.5)		
*p*	0.574		0.636			
LES total length (mm)						
Min.–Max	25.0–45.0	25.0–40.0	25.0–45.0	30.0–40.0	0.885	0.271
Mean ± SD	34.0 ± 6.04	31.33 ± 3.52	34.33 ± 6.51	33.0 ± 4.55		
*p*	0.027*		0.262			
Abdominal LES length (mm)						
Min.–Max	0.0–30.0	0.0–35.0	0.0–30.0	10.0–30.0	0.902	0.838
Median (IQR)	20.0 (2.50–25.0)	20.0 (15.0–25.0)	15.0 (10.0–22.5)	20.0 (15.0–25.0)		
*p*	0.390		0.089			
LES complete relaxations%						
Min.–Max	70.0–100.0	0.0–100.0	80.0–100.0	80.0–100.0	0.828	0.253
Mean ± SD	95.33 ± 9.15	84.67 ± 25.32	96.0 ± 7.37	92.67 ± 7.99		
*p*	0.052		0.238			
LES residual pressure (mmHg)						
Min.–Max	0.0–5.0	0.0–17.0	0.0–4.0	1.0–6.0	0.389	0.567
Median (IQR)	2.0 (1.0–2.50)	4.0 (2.50–6.0)	2.0 (1.0–3.0)	4.0 (3.0–4.50)		
*p*	0.012*		0.006*			
LES duration of relaxation (s)						
Min.–Max	6.0–10.0	5.0–11.0	6.0–11.0	6.0–11.0	0.572	0.654
Mean ± SD	8.47 ± 1.51	8.60 ± 1.72	8.13 ± 1.68	8.33 ± 1.50		
*p*	0.634		0.189			
Peristaltic wave amplitude (mmHg)						
Min.–Max	66.0–155.0	0.0–91.0	72.0–155.0	43.0–143.0	0.714	0.003*
Mean ± SD	98.20 ± 29.34	52.93 ± 22.60	102.0 ± 26.85	84.47 ± 30.46		
*p*	< 0.001*		0.121			
Normal peristaltic waves morphology%						
Min.–Max	90.0–100.0	0.0–100.0	80.0–100.0	80.0–100.0	0.410	0.249
Mean ± SD	99.33 ± 2.58	86.67 ± 25.26	98.0 ± 5.61	94.67 ± 7.43		
*p*	0.075		0.173			
Peristaltic wave velocity (cm/s)						
Min.–Max	2.50–4.80	0.0–4.70	2.50–4.10	2.80–4.20	0.465	0.593
Mean ± SD	3.63 ± 0.90	3.29 ± 1.20	3.43 ± 0.52	3.47 ± 0.46		
*p*	0.306		0.405			

*IQR* interquartile range, *SD* standard deviation, *Min.* minimum, *Max.* maximum, *p1 p*-value for comparing between the two studied groups for preoperative parameters, *p2 p*-value for comparing between the two studied groups for postoperative parameters

*Statistically significant at *p* ≤ 0.05

**Fig. 6:**
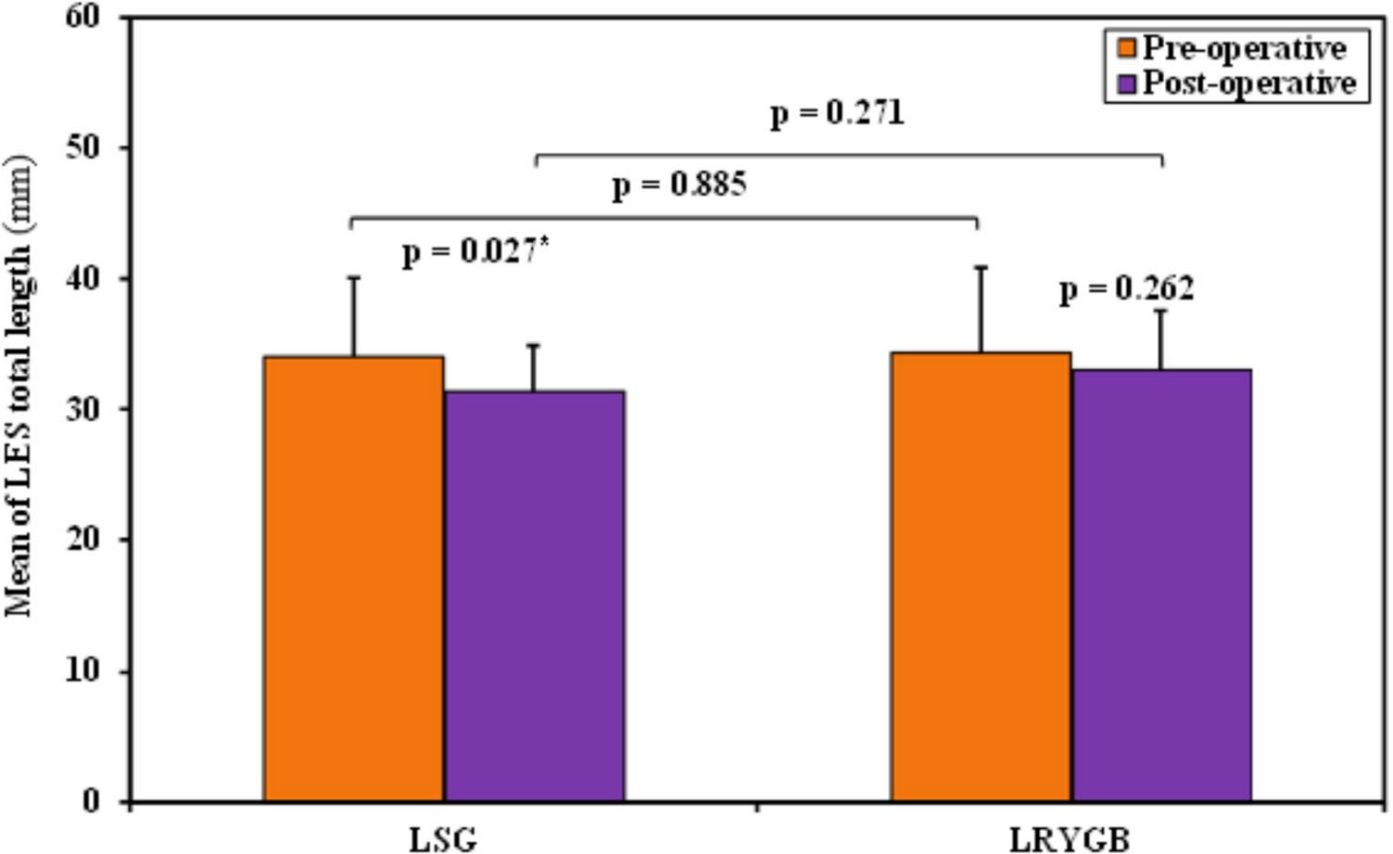
Comparison between the two studied groups according to LES total length

**Fig. 7:**
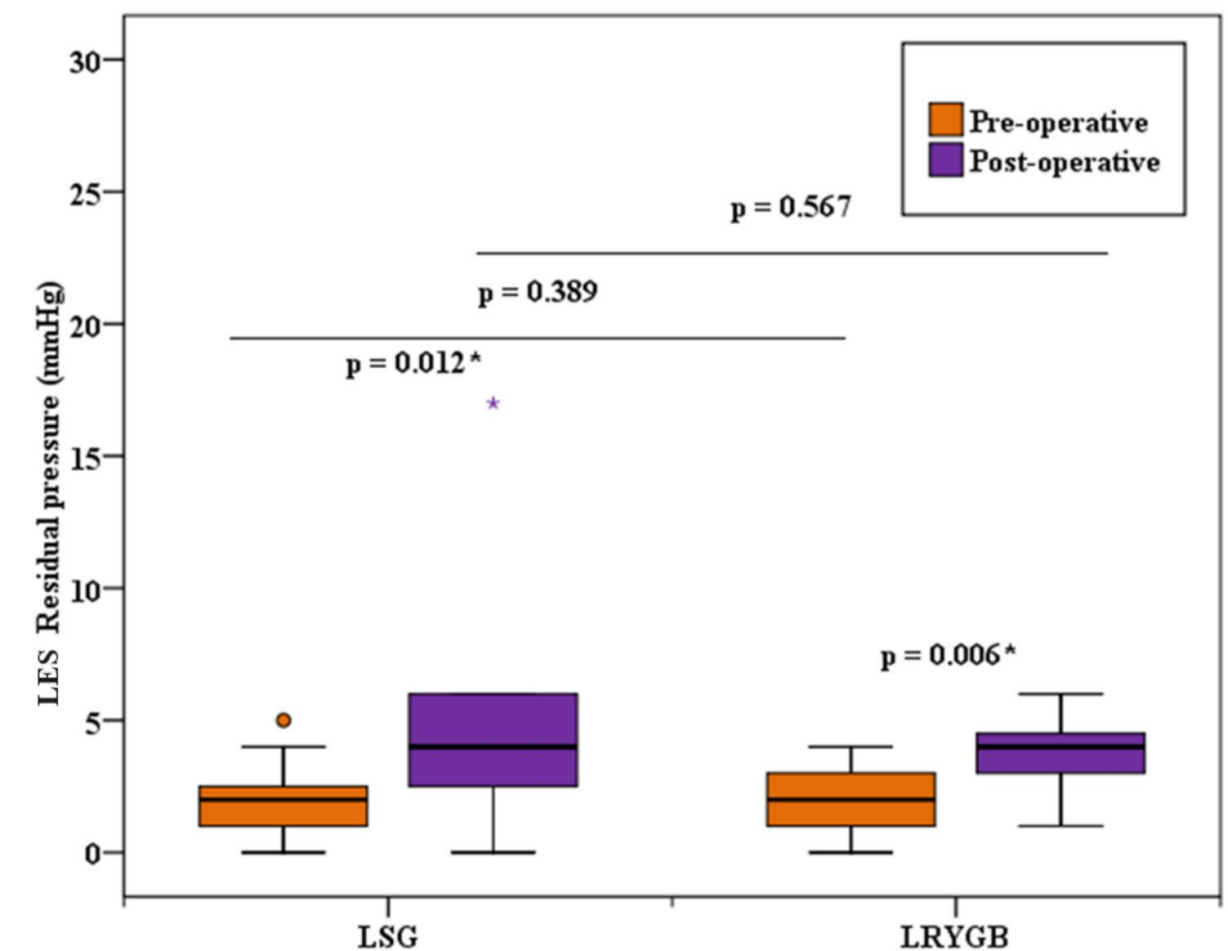
Comparison between the two studied groups according to LES residual pressure

**Fig. 8:**
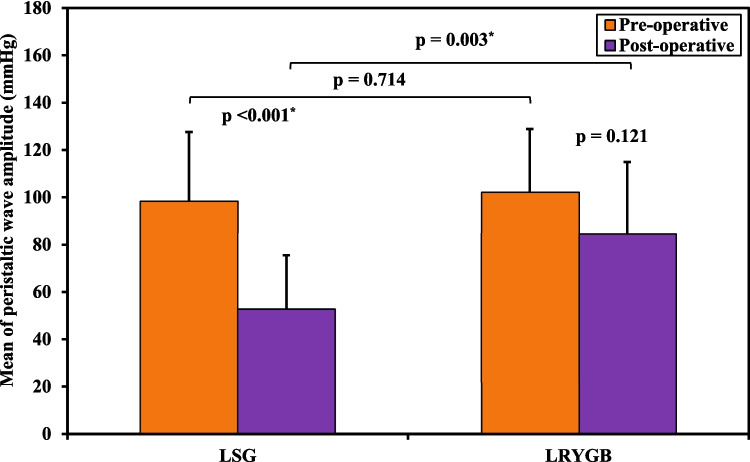
Comparison between the two studied groups according to peristaltic waves’ amplitude

The comparison between the two studied groups according to LES total length, LES residual pressure, and esophageal peristaltic waves’ amplitude is shown in Figs. 6, 7, and 8.

## Discussion

Bariatric surgery is an effective treatment for weight loss and managing obesity-related conditions like GERD. However, its impact on esophageal function varies depending on the type of surgery performed. While weight loss generally improves GERD symptoms, the outcomes differ among surgical procedures. LRYGB is often preferred for patients with both obesity and severe GERD due to its potential to alleviate reflux symptoms. In contrast, LSG may not consistently improve GERD and could, in some cases, worsen reflux symptoms. Preoperative assessment is essential, as no single procedure suits all patients. Some surgeons consider GERD a contraindication for LSG due to the risk of exacerbating reflux [11].

Studies examining the relationship between GERD and esophageal function following LSG and LRYGB have shown considerable variability in methodologies and outcomes, often relying on symptom questionnaires rather than objective assessments. This study aimed to address this gap by utilizing advanced diagnostic tools such as esophageal manometry and MII-pH monitoring to assess GERD and esophageal function. These technologies provide a more detailed understanding of changes in esophageal motility, pressure, and acid exposure post-surgery, offering valuable insights into the effects of LSG and LRYGB on GERD in patients with severe obesity.

The SLEEVEPASS trial [12] (240 patients) and the SMBOSS study [13] (217 patients) are the largest randomized clinical trials comparing LSG and LRYGB, with findings similar to ours. In our study, both groups showed significant postoperative reductions in weight and BMI, with the LRYGB group showing lower postoperative values. The PEWL was higher in the LRYGB group (50.48 ± 8.13%) than in the LSG group (36.21 ± 10.64%), consistent with other studies [14, 15] and a longer-term follow-up may help determine if these differences result from loss of effect of sleeve gastrectomy or from greater weight loss after gastric bypass. Both groups showed significant improvements in GERD-related symptoms and QOL, with no significant differences. The SLEEVEPASS trial also reported greater PEWL in LRYGB (57%) compared to LSG (49%) at 5 years, and both studies reported significant improvements in QOL.

Raj et al. [16] studied 77 patients and found improvements in weight-related parameters 6 months after both LSG and LRYGB, with no significant differences in GERD symptoms between the two groups. Similarly, in our study, GERD symptoms in the LSG group remained stable pre- and post-operation, aligning with Del Genio et al. [17], who found no change in reflux-related symptoms in 25 patients over a median of 13 months. Gadiot et al. [18] reported that 7% of asymptomatic patients developed de novo GERD after LSG, while 96% of those with preoperative GERD had symptom resolution after 5–8 years. In our study, four (26.7%) patients had post-surgery GERD, in whom symptoms resolved in two (50% of the affected). We also had two (13.3%) patients who continued to have GERD and two (13.3%) who developed de novo GERD.

In our LRYGB group, GERD symptoms significantly decreased. Preoperatively, eight patients (53.3%) experienced GERD, but postoperatively this number dropped to one case (6.7%), reflecting an 87.5% resolution of symptoms, with no new cases of de novo GERD. These results align with the bariatric outcomes longitudinal database (BOLD) database, where Pallati et al. [19] studied 22,830 patients, showing a 41% improvement in GERD symptoms and a 9.2% rate of de novo GERD after LSG, while for RYGB, GERD improved in 56.5% of patients. Studies also found RYGB improved GERD by > 90%, with 0–7% de novo GERD [20, 21]. Overall, no significant differences in GERD symptoms were found between the LSG and LRYGB groups pre- or post-surgery.

Clinically, both patient groups in our study reported a significant decrease in reflux symptoms post-operation, as measured by the GERD-HRQL questionnaire, with no difference between the groups. This aligns with a study of 128 patients assessing QOL after 1 year post-LSG and LRYGB using seven questionnaires, which showed similar significant improvements in scores between baseline and 12 months, except for the GerdQ score, which showed a difference between the groups [22]. Other prospective studies confirmed this improvement in scores after both procedures [5, 23]. The BOLD review showed the greatest GERD score improvement in LRYGB patients, followed by LSG [19].

Our study found that PPI usage in the LSG group decreased modestly from 40 to 26.7% postoperatively, while in the LRYGB group, PPI usage significantly dropped from 53.3 to 6.7%. Proton pump inhibitors were prescribed regardless of esophagitis detection, as GERD can occur without visible mucosal changes, which affect about 50% of GERD patients [24]. However, there were no statistically significant differences in PPI usage between the two groups.

Comparing with other studies, Himpens et al. [25] reported 75% resolution of GERD in LSG patients, but 21.8% developed new GERD cases within the first year. A French retrospective study showed over 50% of patients required PPIs during the first year after LSG [26], and Braghetto et al. [27] reported 57.2% of patients using PPIs due to reflux symptoms.

Conversely, studies on LRYGB consistently reported a reduction in PPI usage. Barr et al. [28] noted a 70% decrease 1 year after LRYGB, while Frezza et al. [29] found a reduction from 44 to 9% at 12 months in 152 patients. Parmar et al. [30] observed that 80% of patients who converted from LSG to LRYGB stopped GERD medications. However, Holmberg et al. [31] found persistent GERD in 48.8% of 2454 RYGB patients after 4.6 years, requiring acid-suppressing medications for more than 6 months in nearly half within 2 years.

In our LSG cases, EGD showed a decrease in esophagitis from 66.7 to 26.7%, with 20% having LA-A and 6.7% having LA-B esophagitis, comparable to another study reporting 26.3% with LA-A and 15.8% with LA-B; however, that study also noted a 42.1% increase in esophagitis post-sleeve gastrectomy [32]. De novo GERD signs were seen in 13.3% of cases, consistent with other studies showing new esophagitis ranging from 6.5 to 66.7% [27, 33, 34]. Sharma et al. [5] found post-LSG esophagitis in 25% of patients. In contrast, for LRYGB cases, EGD revealed a reduction in esophagitis from 53.3 to 6.7%, with no new GERD findings, aligning with other studies that showed esophagitis decreased from 42 to 4% post-LRYGB [35], with some cases persisting at 20% after 1 year (yet with 11% improved) [36]. One case (6.7%) of Barrett’s metaplasia occurred after LSG, falling between Braghetto’s 1.2% and Genco’s 17.2% [37, 38]. These differences might be due to the strict endoscopic follow-up of cases, low severity of GERD (according to LA classification), and the use of PPIs. No metaplasia cases were observed after LRYGB, similar to a study with a 3-year follow-up [23].

In our study, barium swallow studies showed a decrease in reflux signs from 33.3 to 13.3% after LSG and from 66.7 to 0% after LRYGB. A review by Howard et al. [39] found that 18% of patients had GERD signs post-LSG, with a 40% PEWL and a mean follow-up of 8 months.

Gastric morphology is another factor contributing to the progression of esophagitis after bariatric surgery. Dias da Silva et al. [40] found that 28.3% of patients with esophagitis post-LSG had abnormalities in the gastric tube, including gastric dilation, twist, neo fundus formation, and hiatal hernia. In our study, 20% and 13% of patients had hiatal hernias after both surgeries, respectively. Despite a significant reduction in hernia cases (postoperatively in both groups by 50% and 33.3%, respectively) due to weight loss, the size of the gastric tube was not measured (due to the lack of computed tomography (CT) scans done, which are more accurate than barium studies). The correlation between hiatal hernias and GERD symptoms was noted, and some studies suggest that concurrent hernia repair during bariatric surgery, especially for small hernias, might not always be beneficial and could introduce complications and that is what we believed in. Kothari et al. [41] found that many surgeons avoid hernia repair during LRYGB, while Snyder et al. [42] reported no impact on GERD symptoms from repairing small hiatal hernias (< 4 cm) with LSG. All preoperative hiatal hernias in both our groups were small (< 4 cm), which did not need definitive repair.

Accelerated gastric emptying after LSG was reported by Melissas [42], but Bernstine et al. [43] did not find the same results. Our study observed no abnormal gastro-duodenal emptying and no delayed gastrojejunal emptying after LRYGB. This aligns with Näslund et al. [44], who also did not find delayed emptying after LRYGB, except in one patient with anastomotic stenosis. Variations in results may be due to differences in surgical techniques and the lack of scintigraphy.

MII-pH monitoring has significantly enhanced the evaluation of GERD by detecting a greater number of reflux episodes compared to traditional 24-h pH monitoring. This advancement is achieved by assessing both the physical and chemical properties of the refluxate. The incidence of new-onset GERD following bariatric surgery varies, influenced by factors such as the duration of follow-up and the criteria used for diagnosis. Currently, MII-pH is regarded as the leading method for diagnosing and managing GERD, as it identifies acidic, weakly acidic, and alkaline refluxes, and differentiates between liquid, gas, and mixed refluxes, thereby offering a comprehensive characterization of the condition.

Studies on LSG’s impact on acid exposure show mixed results: some indicate a significant increase in total acid exposure after 12 months [45], while others report a decrease within the same timeframe [46]. Although LRYGB is considered the optimal treatment of GERD in patients with severe obesity [47], it may not always fully resolve GERD symptoms [25, 48]. Some studies have reported that up to 22% of patients who undergo LRYGB continue to experience GERD symptoms postoperatively [29].

Unlike previous studies reporting significant increases in AET% and DMS after LSG [49, 50], our study found only a mild, non-significant rise in these parameters, with AET% increasing from 6.7 to 46.7% and DMS from 6.7 to 20%. This result is consistent with a prior trial involving 37 patients, where no significant changes in DMS or AET% were noted, and DMS increased in 18.9% of cases [46]. Similarly, a recent study of 30 patients with a 1-year follow-up reported a non-significant rise in AET% and DMS, with QOL remaining unaffected [51]. Additionally, while there was a non-significant decrease in the total number of reflux episodes and acidic refluxes, this trend aligns with another study showing a stable DMS and a non-significant reduction in reflux episodes and acid exposure after 1 year [32]. These findings may be influenced by continued PPI use despite recommendations to discontinue them 15 days before assessment.

In our LRYGB group, there was a notable reduction in the DMS post-operation, with pathological DMS decreasing from 20 to 6.7%. However, the AET% remained largely unchanged. These findings are consistent with previous studies which also reported significant improvements in DMS after LRYGB [20, 52], with postoperative pathological DMS observed in only 9% and 12.5% of patients in other studies [16, 23]. We also observed a significant reduction in the total number of reflux episodes and acidic refluxes, which decreased from 20% preoperatively to 6.7% postoperatively. These results align with previous research showing significant improvements in various pH-based parameters, such as the percentage of time with esophageal pH < 4, the total number of reflux episodes, and the number of acidic reflux episodes [23, 53].

The study of non-acidic reflux in bariatric surgery is limited. Our research found no significant changes in the number of non-acidic reflux episodes within or between LSG and LRYGB groups, aligning with another group’s findings [16]. This lack of significance is likely because alkaline reflux is prevented by the Roux-en-Y anastomosis distal to the gastrojejunostomy.

For weakly acidic reflux, our study showed no significant changes overall, though there was a minor decrease in the LSG group and an increase in cases exceeding normal levels from 6.7 to 20%. This contrasts with Chern et al. [54], who reported a significant increase in weakly acidic reflux after LSG due to reduced gastric acidity after resection; however, our study did not observe the significant decrease in LES pressure noted by theirs.

In the LRYGB group, we saw a slight rise in weakly acidic reflux episodes, but the percentage of cases exceeding normal levels remained stable at 6.7%. This differs from Morino’s group [55], who found that 75% of patients had weakly acidic reflux and 44% developed esophagitis in their 5-year follow-up. Their results suggested that abnormal motility in the Roux limb, supported by a recent study on electrical activity and manometry, might contribute to these findings [56].

Esophageal manometry offers both quantitative and qualitative assessments of esophageal pressure and peristaltic coordination, including measurements of the LES. However, findings regarding manometric changes following bariatric surgery remain inconsistent. In our study comparing both groups, LES parameters were similar; however, alterations in impaired motility appeared to influence reflux.

Our study found no significant changes in LES basal pressure following LSG. Preoperatively, 33.3% of patients had LES pressures below the normal range, which increased slightly to 40% postoperatively. This result is consistent with Del Genio et al.’s study of 25 patients [17], which showed no significant changes in LES pressure and an increase in ineffective motility 1 year after LSG. Similarly, Rebecchi et al. [46] reported no changes in LES pressure. In contrast, Braghetto et al. [27] observed a significant decrease in LES pressure in 85% of patients 6 months post-LSG, attributed to partial sectioning of the sling fibers. Burgerhart et al. [57] also noted reduced LES pressure 3 months after LSG, despite stable peristalsis. Petersen et al. [58] suggested that higher LES pressure post-LSG might protect against GERD, potentially due to variations in surgical technique. These findings suggest that isolated LSG may not significantly impact the LES, highlighting the importance of surgical technique, especially in dissection near the gastroesophageal junction.

The LRYGB group showed no significant changes postoperatively in LES basal pressure which was below the normal range in 13.3% of patients and decreased to 6.7% after surgery. Similarly, previous studies have reported non-significant changes in LES pressure at both the same and longer follow-up periods [16, 55].

In our study, the total LES length tended to decrease after LSG and showed a non-significant shortening after LRYGB. The abdominal LES length remained unchanged after LSG and slightly increased post-LRYGB, but these changes were not statistically significant between the groups. The observed shortening of the LES following LSG might be due to increased intragastric pressure compressing the LES, similar to effacement from gastric distention. Despite significant weight loss in both groups, the abdominal LES length did not reduce. Other studies have reported conflicting results, with one showing an increase in LES length after LSG [59] and another showing an increase after LRYGB [16].

Interestingly, the median LES residual pressure nearly doubled postoperatively in both groups, with only one case (6.7%) in group A exceeding the normal range. This is contrary to other studies where LES residual pressure slightly decreased [54, 60]. The increased intragastric pressure and reduced distensibility of the sleeve might heighten tension at the gastroesophageal junction. Additionally, dissection of the phreno-esophageal membrane for stapler placement near the angle of His may disrupt the esophageal sphincter’s integrity, affecting outcomes in gastric bypass [23].

The study found a significant difference in peristaltic wave amplitude between the two groups postoperatively. In the LRYGB group, peristaltic amplitude decreased slightly but stayed within normal ranges for all patients and had no clinical significance. In contrast, the LSG group showed a more pronounced decline in esophageal contractility, with one patient (6.7%) falling below the normal range postoperatively. This decline may lead to ineffective clearance of regurgitated gastric contents and stasis, possibly as a compensatory mechanism for the weakened LES. While some studies reported no significant changes in esophageal wave amplitude post-sleeve gastrectomy [46, 49], findings from two Italian studies with 25 and 21 patients, respectively, indicated a significant increase in ineffective esophageal motility following LSG [17, 32]. Conversely, studies on Roux-en-Y gastric bypass consistently found no severe impairment in esophageal peristaltic amplitude [48, 52].

A patient from the LSG group was diagnosed with type II achalasia, as confirmed by high-resolution manometry (HRM). This diagnosis was based on a LES basal pressure of 41 mmHg, a residual pressure of 17 mmHg, failure of complete relaxation, and absent peristaltic waves, which prevented assessment of amplitude, velocity, and morphology. Such a finding is rare after LSG, as noted in the systematic review by Crafts et al. and he was scheduled for a Heller myotomy with conversion surgery [61].

Our study is prospective and integrates both subjective and objective parameters for assessment. Additionally, since no significant preoperative differences were observed between the groups, this design helps minimize potential biases. The primary limitations of this study include the relatively short follow-up period and the limited, non-randomized sample size. The study faced challenges in recruitment, as many patients declined to undergo the minimally invasive investigations. Additionally, CM was utilized instead of the more advanced HRM technology.

## Conclusions

Both LSG and LRYGB have shown effective weight loss and improvements in QOL at 1 year post-surgery. In patients with obesity with pre-existing GERD, LSG often alleviates symptoms and controls reflux. De novo GERD following LSG is uncommon and typically linked to ineffective esophageal motility rather than changes in LES pressure. Both LSG and LRYGB remain viable options for managing obesity in GERD patients.

Advanced diagnostics, such as manometry and MII-pH monitoring, offer important metrics for assessing gastroesophageal function, particularly when standard methods like endoscopy or barium studies do not explain symptoms. Larger randomized studies with longer follow-up periods and incorporating technologies like high-resolution impedance manometry (HRIM) are needed to further evaluate postoperative gastroesophageal function and symptom resolution in bariatric patients.

## Data Availability

No datasets were generated or analysed during the current study.
